# Simulation and real-life implementation of UAV autonomous landing system based on object recognition and tracking for safe landing in uncertain environments

**DOI:** 10.3389/frobt.2024.1450266

**Published:** 2024-10-18

**Authors:** Ranjai Baidya, Heon Jeong

**Affiliations:** ^1^ Kpro System, Namyangju-si, Republic of Korea; ^2^ Department of Fire Service Administration, Chodang University, Muan-gun, Republic of Korea

**Keywords:** autonomous landing, deep sort, distance transform, intelligent autonomous system, obstacle avoidance, object detection, PID control, YOLOv5

## Abstract

The use of autonomous Unmanned Aerial Vehicles (UAVs) has been increasing, and the autonomy of these systems and their capabilities in dealing with uncertainties is crucial. Autonomous landing is pivotal for the success of an autonomous mission of UAVs. This paper presents an autonomous landing system for quadrotor UAVs with the ability to perform smooth landing even in undesirable conditions like obstruction by obstacles in and around the designated landing area and inability to identify or the absence of a visual marker establishing the designated landing area. We have integrated algorithms like version 5 of You Only Look Once (YOLOv5), DeepSORT, Euclidean distance transform, and Proportional-Integral-Derivative (PID) controller to strengthen the robustness of the overall system. While the YOLOv5 model is trained to identify the visual marker of the landing area and some common obstacles like people, cars, and trees, the DeepSORT algorithm keeps track of the identified objects. Similarly, using the detection of the identified objects and Euclidean distance transform, an open space without any obstacles to land could be identified if necessary. Finally, the PID controller generates appropriate movement values for the UAV using the visual cues of the target landing area and the obstacles. To warrant the validity of the overall system without risking the safety of the involved people, initial tests are performed, and a software-based simulation is performed before executing the tests in real life. A full-blown hardware system with an autonomous landing system is then built and tested in real life. The designed system is tested in various scenarios to verify the effectiveness of the system. The code is available at this repository: https://github.com/rnjbdya/Vision-based-UAV-autonomous-landing.

## 1 Introduction

Unmanned Aerial Vehicles (UAVs) are gaining wider acceptance due to their advantages over manned flights (Mohsan et al., 2023). They are versatile and used in fields such as transportation, search and rescue, military, surveillance, agriculture, and delivery (Mohsan et al., 2023). Additionally, they are also cost-effective and require fewer resources to operate and maintain. In scenarios like disaster response, firefighting, and hazardous material spills, UAVs are safer because no human lives are at risk. Furthermore, autonomous UAVs can transform various sectors, making them a topic of increasing interest (Hassanalian and Abdelkefi, 2017).

**FIGURE 1:**
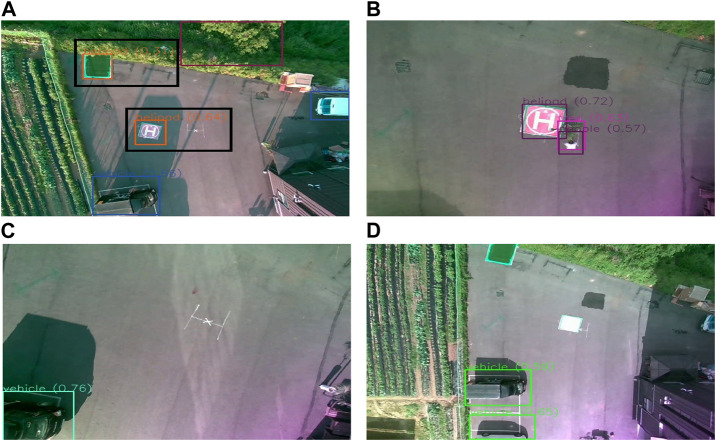
Exhibition of different undesirable scenarios during vision-based autonomous landing in UAV: **(A)** Object detection algorithm misclassifying another object as a landing pad (black box is manually drawn to help better identify the detections), **(B)** presence of obstacle nearby the landing pad, **(C)** absence of the visual markers denoting the landing pad, **(D)** the present landing pad is not properly identified due to reflection of light on the pad.

Achieving full autonomy in UAVs requires a robust control system (Chen et al., 2009). One of the notable challenges to achieving full autonomy is the ability to land at a marked spot. Even a minor error during UAV movement can cause significant damage to the UAV itself and its surroundings, necessitating research into methods to reduce these risks. Moreover, implementation of autonomous systems outside of a controlled environment into real-life scenarios could be much more challenging due to the unpredictability of the world. So, it is necessary to make these systems robust to some undesirable situations in the real world. Some of these situations are detection of multiple landing pads, the presence of obstructive objects near the landing target or the inability to find the visual marker indicating the landing target. These scenarios can be seen in Figure 1. Figure 1A shows the output of an object detection algorithm where two landing targets are identified by the object detection algorithm, with one of them being wrongly classified. Figure 1B shows a scenario of an obstacle in unsafe proximity of the landing target. Figures 1C,D both show conditions where the landing pad is not detected at all. While in Figure 1C the landing pad is fully absent, in Figure 1D the landing pad is not properly visible due to reflection of light. These kinds of undesirable situations can be considered explicitly to build systems around them to deal with these cases.

This paper first presents a simple method to perform autonomous landing with a quadrotor UAV using visual cues on top of a designated marker. Then the discussed undesirable scenarios are accounted for to increase the reliability. The considered scenarios are:

• The presence of multiple markers indicating the landing pad.

• The presence of obstacles near the marker.

• The inability to detect the designated visual marker or the absence of the marker altogether.

The overall system is built based on the Proportional-Integral-Derivative (PID) control, the You Only Look Once (YOLOv5) algorithm for object detection using a camera input, the DeepSORT algorithm for tracking, and a simple algorithm based on distance transform to find the open space. Two PID controllers generate control values for the left/right and forward/backward movement of the UAV, which utilize the discrepancies between the center of the landing target and the center of the image frame obtained from the camera pointed toward the ground. We trained the YOLOv5 algorithm on images of classes such as “people”, “vehicle,” “helipad,” and “tree” for recognizing the visual marker and the obstacles. Before deploying the system in autonomous flights some initial tests are performed. First, we evaluate the capability of the system to accurately measure the distance between two points in a camera frame. To do this, we manually fly the UAV while capturing camera frames and the altitude of the UAV at the corresponding time. The distance between a point and a visual marker is then measured using a measuring tape. Then we calculate corresponding distances. After ensuring the difference between the measured distance and calculated distance is negligible, we perform a simulation of the landing process using the Software in the Loop simulator of Ardupilot. After finetuning the system in both ways, we deployed the system in the real world. The final system successfully performs autonomous landing in the designated spot in case of absence of any unfavourable scenarios and in a safe spot which may or may not be the designated spot in presence of some uncertainty. The main contributions of this paper can be listed as follows:

• Suggest a robust algorithm for safe vision-based autonomous landing of quadrotor UAVs in undesirable scenarios.

• Designing initial tests and simulation environment for the initial testing of the system in undesriable landing scenarios.

## 2 Related works

The escalating demand for autonomous UAVs has led to several research efforts toward designing autonomous landing and obstacle avoidance systems for these vehicles. These systems are necessary in multiple academic and industrial fields, including search and rescue, agriculture, and package delivery. Various works in the fields of autonomous landing, obstacle avoidance, and object detection have been performed.

### 2.1 Autonomous landing

Several previous attempts have been made to enhance the capabilities of autonomous UAVs for autonomous landing. One such work utilized a combination of Canny edge detection; Hough transform and Hu invariant moment to detect the landing platform (Tsai et al., 2006) and then perform the attitude estimation. They performed testing on a “T-Wing“ V.T.O.L, and were able to get root mean square errors of 4.8°, 4.2°, and 4.6° during attitude estimation. Another work utilizing classical image processing technique was (Saripalli et al., 2002). This work utilized Hu invariant moment to resolve the position along with differential Global Positioning System (GPS) to perform the autonomous landing on an UAV helicopter, while getting an average orientation error of 7° (Fan et al., 2008). deployed a system to perform noise removal using median filtering, then enacted imaged segmentation using a fixed threshold on Zernike moments to find the landing platform. Their experiments demonstrated that Zernike moments are superior to Hu moments in identifying the landing point robustly, even in the event of landing pad rotation. They also demonstrated R.M.S error of 4.21 cm, 1.21 cm, and 0.56° in x-position, y-position and orientation respectively (Wenzel et al., 2011). presented a method to trace a unique arrangement of infrared lights on a platform using an infrared camera to locate the landing point. While this method was successful 90% of the time it was only suitable for indoor use. Another work utilized optical flow estimation to track a textured platform for non-linear controller of vertical take-off and landing (VTOL) UAV equipped with a simple sensors setup of only camera and inertial measurement unit (IMU) (Herissé et al., 2011; Lee et al., 2012) utilized image-based vision servoing (IBVS) to track a landing platform in a two-dimensional image and estimated the required velocity to be used as an input to the adaptive sliding mode controller. The disadvantage of this work was its reliance on a single marker to mark the landing platform, making it susceptible to loss of the marker. There has been other work to solve the problem of losing the marker by using fish-eye cameras. However (Kim et al., 2014), utilizes simple color-based tracking, which is highly susceptible to false detections, even more so in the outdoor environments. Also (Baca et al., 2017) utilizes prior knowledge of the expected path of the target. Additionally, many works also utilize fiducial markers for a vision-based navigation during the landing (Araar et al., 2017). has an average error of 13 cm from the landing pad but is only tested in indoor environments (Salagame et al., 2022). fails when there is a large change in speed or direction.

In recent years, deep-learning algorithms have been increasingly used for landing target detection in vision-based autonomous landing systems (Chen et al., 2017). used faster regional neural networks (Faster R-CNN) to detect the landing pad and obtained an average error of 2.23°, 1.18 cm, 1.31 cm and 1.29 cm while estimating yaw, orientation in *x*-axis, *y*-axis and *z*-axis respectively. In (Nguyen et al., 2018), LightDenseYOLO was used instead of template matching, and the accuracy was further improved by implementing Profile Checker (Truong et al., 2020). suggested utilizing deblur generative adversial network (DeblurGAN) to deal with non-unifrom motion-blurred inputs and furthermore YOLOv2 for the detection of the landing area (Neves et al., 2024). presents a multimodal transformer-based detector for precise UAV landing and a reinforcement learning model for decision-making, achieving high reliability and rapid inference times even in diverse and challenging conditions (Aikins et al., 2024). introduces a robust deep reinforcement learning approach using LSTM networks, called RPO-LSTM, which significantly enhances UAV autonomous landing on moving platforms under partial observability, outperforming existing methods in challenging conditions with sensor impairment and environmental noise.

### 2.2 Obstacle avoidance

Numerous research has been performed for detecting and avoiding the obstacles around the UAV. Multiple such research is based on traditional methods (Mori and Scherer, 2013). detect obstacles based on the relative size changes of image patches. They utilize speeded-up robust features (SURF) for feature matching alongside template matching to compare relative obstacle sizes with different image spacing. Many works also utilize optical flow for performing obstacle avoidance (Souhila and Karim, 2007). utilizes optical flow for obstacle avoidance in an autonomous mobile robot (Muratet et al., 2005). utilizes optical flow alongside inertial information to avoid collisions in an autonomous helicopter (Peng et al., 2016). combine the depth cues with optical flow to detect the obstacles in a quadrotor system (Müller et al., 2024). introduce an ultrasonic sensor-based system inspired by bats to enable nano-drones to navigate autonomously and avoid obstacles (Zhang et al., 2024). propose a lightweight CNN depth estimation network using Channel-Aware Distillation Transformer (CADiT) inspired by knowledge distillation for obstacle avoidance in nano-drones with limited computing power.

### 2.3 Object detection

Conventional approaches for object detection utilize feature extraction methods such as Histogram of Oriented Gradients (HOG) (Dalal and Triggs, 2005) or Scale Invariant Feature Transform (SIFT) (Lowe, 2004), which require a significant amount of manual input and time. In recent years, research on object detection has shifted towards deep learning approaches that can be broadly categorized as anchor-based or anchor-free object detection architectures, based on their use of pre-defined sliding windows.

Anchor-based methods involve the classification of object boxes into distinct bins, followed by box rectification. Region-CNN (RCNN) (Girshick et al., 2014), Spatial Pyramid Pooling (SPP) network (He et al., 2015), Fast RCNN (Girshick, 2015), Single Shot multibox detector (SDD) (Liu et al., 2016), the You Only Look Once series of models: YOLOv1 (Redmon et al., 2016), YOLOv2 (Redmon and Farhadi, 2017), YOLOv3 (Redmon and Farhadi, 2018), YOLOv4 (Bochkovskiy et al., 2020), YOLOv5 (Jocher et al., 2020) are some examples of anchor-based methods. Conversely, anchor-free methods do not engage with computations pertaining to anchor boxes, instead employing alternative methodologies. Fully Convolutional One stage (FCOS) (Tian et al., 2019), Feature Selective Anchor Free Module (FSAF) (Zhu et al., 2019), YOLOX (Ge et al., 2021) are some examples of the anchor-free methods. Furthermore, some attention based methods such as (Ouyang et al., 2023) are also used for object detection (Ouyang et al., 2023). propose Efficient Multi-scale Attention (EMA) module that improves feature representation and computational efficiency by grouping channel dimensions and using cross-dimension interaction, achieving superior performance in image classification and object detection tasks compared to recent methods. Additionally, object detection is also performed in infrared images (Zhang et al., 2021). introduce a backbone network, called Deep-IRTarget,for combining frequency and spatial feature extractors with a dual-domain resource allocation model, significantly enhancing object detection in infrared images.

Object detection in general images is much different than object detection in the images captured from the field of view of UAVs. In the context of UAV-captured images, object detection poses significant challenges, chiefly due to the wide variation in shape and size of the objects of interest, as well as the potential for a high number of objects to be detected. Additionally, computing resources on UAVs are inherently limited, further complicating the task of object detection. Works like Peele (Ozge Unel et al., 2019), ClutDet (Yang et al., 2019), and DMNet (Li et al., 2020) focus on the object’s size and deploy coarse-to-fine frameworks. M-CenterNet was also introduced to deal with minimal sized objects in frames captured from aerial devices (Ding et al., 2021). Transformer Prediction Head YOLOv5 (TPH-YOLOv5) modified YOLOv5 architecture to accommodate the needs of object detection in UAV images (Zhu et al., 2021). TPH-YOLOv5 added an extra prediction head to solve the issue of object detection of small objects and employ self-attention mechanism in the prediction head. Additionally, convolutional attention model (CBAM) has also been utilized in TPH-YOLOv5 to locate the region of interest in scenarios characterized by densely packed objects. Another work further modified the architecture of TPH-YOLOv5 to using ConvMixers instead of transformers in the prediction heads, to make the architecture more computationally efficient (Baidya and Jeong, 2022). Finally (Zhao et al., 2023), introduce YOLOv7-sea, an enhanced object detection algorithm incorporating additional prediction heads and attention modules, along with data augmentation techniques, achieving improvement over YOLOv7 in detecting small targets and reducing sea surface interference in maritime search and rescue scenarios.

## 3 Methods

In this section, we will discuss the solutions to the undesirable scenarios that the system deals with, the overall algorithm and the individual components used in the system. The content discussed in this section will be in the following order: undesirable scenarios, their solutions and the overall algorithm, YOLOv5 and DeepSORT, meters per pixel calculation, PID Controller, algorithm to find the empty space.

### 3.1 Undesirable scenarios, their solutions and the overall algorithm

We propose different methods to deal with the undesirable but highly plausible scenarios during vision-based autonomous landing. Here we will discuss how the targeted undesirable scenarios are dealt with.

• False detection of landing pad: Object detection techniques may not always be reliable and sometimes there may be instances where false detections are encountered. The system is designed such that it only considers the detections of the landing pad where the confidence score is above 50%, the probability of failure of the system due to such false detections are reduced.

• Absence of a marker denoting landing pad: There are possibilities that the visual marker denoting the landing pad is not detected by the object detection algorithm. This could be caused by the reasons such as presence of the landing pad in an area out of field of view of the camera, obstruction of the landing pad, absence of the helipad altogether or sometimes even the inability of the object detection algorithm to detect the visual marker. In any of such scenarios, the designed system is capable to land in a safe landing spot. After reaching the end of the mission, in case the landing pad is not recognized, the UAV is elevated slowly to 5 m higher than the altitude at the end of the mission. If the landing pad is recognized at any point while raising the altitude of the UAV then the UAV proceeds towards landing in the landing pad, otherwise a nearby alternate safe landing spot is considered based on the algorithm described in Section 3.5.

• Presence of multiple landing pads: The object detection algorithm can also sometimes detect two landing pads in a single frame due to actual presence of such landing pads or due to some false detection. Our system only considers only the landing detection with the highest confidence and the one closest to the location at the end of the mission. Additionally, landing pad choosen are also kept in track using the Deep Sort algorithm.

• Presence of obstacles nearby the landing target: There are possibilities of presence of obstacles nearby the landing pad, which may move or remain stationary during the landing process. Eitherway, our system can proceed with the safe autonomous landing. Upon detection of obstacles nearby landing spot the system will sound a buzzer so as to notify that the drone is proceeding with landing. While sounding the buzzer, the system waits for 10 s so to observe whether or not the landing pad is devoid of any obstacles. If the landing pad is cleared within 10 s then the system proceeds with landing. Otherwise, the system proceeds to land in an alternate safe landing spot as per the algorithm discussed in Section 3.5.

Algorithm 1 Algorithm to save information regarding the landing pad, obstacles and the alternate safe landing space.

**Input:** Queue storing captured camera frames **“q_frames”**; YOLOv5 model loaded with pre-trained weights **‘yolo’**
**Output:** list of the landing pad detections with their position and confidence scores **“helipad”**; list of obstacle detections with their position and confidence scores **“obstacles”**; position of the largest open space available **“safe”**
**while** landing is not complete **do**
 
pred←yolo(q_frames.get()) ⊳ yolo returns the detections with their class labels, confidence scores, and positions
 
empty←binary image frame with a small border

 **for** each detection **p** in **pred do**
  **if p. class** is one of obstacle class **then**
   obstacles.append(**p**)
   draw rectangular blob of size of **p** on **empty** at the position of **p**
  **else if p. class** is of landing pad **then**
   **helipad**.append(**p**)
 
safe←DistanceTransform(empty)

The overall algorithm for autonomous landing requires information regarding the obstacles, the landing pad and the alternate safe landing space. These information are continually extracted and saved such that they are accessible to the rest of the program. The process of extracting these information and storing them is shown in Pseudo code 1. The YOLOv5 and the Deep SORT algorithm are applied to each frames obtained from the camera. Based on the output of the YOLOv5 algorithm, some alternate safe landing spots are also identified, using the method mentioned in section 3.5. The locations of the landing target detection, the alternate safe landing spot, and the obstacles are saved to a queue, which is accessible by the rest of the program while performing the autonomous landing.

Pseudo code 2 shows the overall landing algorithm. The algorithm initially monitors whether the mission has been completed and whether the autonomous landing can be started. Once the mission is complete, the PID controllers are initialized, and the algorithm searches for the detection of the landing pad in the queue described above. If there are no landing pad detections in the queue, the algorithm raises the altitude of the UAV for 10 s by 0.5 m after each time the detections are not present. Even after that time, if the landing pad is not found, the UAV will proceed to landing to the nearest largest empty area. If the location of the landing pad is found, then the UAV proceeds to land normally. After that, the algorithm continuously searches for obstacles near the landing spot. The algorithm proceeds to land normally if there are no obstacles detected throughout the landing process. In case some obstacles are seen within 1 m of radius of the landing spot, then the UAV will wait for 10 s while sounding an alarm to see if the obstacle will move. When the obstacle moves away from the landing spot, the alogrithm will continue with landing. During the entire process, the algorithm finds the discrepancy between the image frame center and the landing target center. Based on whether this value is large, the algorithm decides whether to lower the altitude of the UAV. If the discrepancy is larger than 2 m, the algorithm only sends the control values generated by the PID controllers for forward/backward and left/right movement of the UAV. Otherwise, the algorithm lowers the altitude of the UAV by 0.5 m while also sending the control values generated by the PID controllers. We consider an altitude of 1 m to be safe to perform normal landing so, once the altitude of the UAV is less than 1m, the algorithm sends “LAND” command to the FC.

Algorithm 2 Algorithm for safe autonomous landing.

**Input:** list of obstacles information **‘obstacles’**; list of landing pad information **‘helipad’**; information of largest open space available **‘safe’**
**while** mission is running **do**
 **if** mission is completed **then**
  Break

PID← PID controller initialization to estimate required drone movement
**Target _is _heli** ←True

**while** UAV is not landed **do**
 **If** UAV altitude is greater than 1 meter
  Get **helipad** and **obstacles** from Algorithm 1
  if **Target _is _heli** is *True* **then**
   **if** helipad is not None **then**
    **if** len (**helipad**) > 1 **then**
     
target←item with highest confidence on helipad

    **else**
     
target←helipad[0]

    
error←discrepancy between camera frame center and target

    
movement←PID(error)

    **if movement** > 2 **then**
     send command to flight controller for horizontal movement
    **else**
     **if** obstacles is not None **then**
      **if** any **o** in **obstacles** is in close proximity to **target then**
       **if** the counter **time_of_wait** is not started **then**
        
time_of_wait←timer to denote the presence of obstacle o

       Wait for obstacle to move away
       **if time_of_wait** > 10 s **then**
        
Target_is_heli←False

      **else**
       send command to FC for horizontal + vertical movement
     **else**
      send command to FC for horizontal + vertical movement
   **else**
    **send** command to flight controller to raise Altitude by 0.2 meter
    **if** alt is greater than altitude at end of mission by 5 meters **then**
     **Target_is_heli** ←False

  **else**
   
error←discrepancy between camera frame center and target

   
movement←PID(error)

   **if movement** > two **then**
    send command to FC for horizontal movement
   **else**
    send command to FC for horizontal + vertical movement
 **else**
  Send LAND command to FC

### 3.2 You only look once version 5 (YOLOv5) and simple online and real-time tracking with a deep metric association (deep SORT)

Our approach utitlizes YOLOv5 for object detection and DeepSORT for object tracking. This combination allows for efficient and accurate dection and tracking of objects in video streams.

The YOLOv5 [31] object detection framework is based on a single shot detection (SSD) approach that processes the entire input image in a single feed-forward pass. The architecture consists of three main components: backbone network, neck, and head. The backbone network, a feature extractor, is responsible for extracting high-level features from the input image. YOLOv5 uses the CSPNet (Wang et al., 2020) architecture as the backbone network, which is an optimized version of the ResNet architecture.

The CSPNet (Wang et al., 2020) is composed of a stem layer, a series of CSP blocks, and a global pooling layer. The stem layer processes the input image and extracts initial features, which are then passed through a series of CSP blocks. The CSP block is a residual block that divides the input features into two branches, one with fewer channels and another with more channels. The low-channel branch is then processed through a series of convolutional layers and concatenated with the high-channel branch, which is processed through a shortcut connection.

The neck connects the backbone network to the head and is composed of a combination of convolutional and pooling layers. The neck uses the SPP (Spatial Pyramid Pooling) (He et al., 2015) module, which divides the input features into different scales and applies max pooling to each scale, allowing the network to capture features at different scales.

The head is responsible for predicting the bounding boxes, class probabilities, and confidence scores of the objects in the input image. The head architecture is composed of multiple convolutional and linear layers that perform the final prediction. The head uses the PAN (Path Aggregation Network) (Liu et al., 2018) module, which aggregates features from different scales and refines them to produce the final prediction.

YOLOv5 introduces several new techniques, such as anchor-free detection, that eliminates the need for anchor boxes, which improves the accuracy of object detection. Additionally, YOLOv5 uses a hybrid approach for training, which combines both supervised and unsupervised techniques, resulting in improved model generalization and robustness.

To complement the YOLOv5’s detection capabilities, we incorporate the Deep SORT (Wojke et al., 2017) algorithm for tracking the detections. This integration creates a seamless pipeline from detection to tracking, enhancing the overall performance of or system.

DeepSORT builds upon the SORT algorithm by incorporating a deep convolutional neural network to extract discriminative features from raw image data. These features contain essential information about the object’s appearance and spatial information; hence, they tend to be reliable representations of these objects during tracking. Then Kalman filter-based approach enables the data association process. Here, the features extracted in the previous step and the object’s state estimations are integrated by factoring motion, position uncertainties, and the previously predicted state. The tracks from the previous and the current steps are matched using the intersection over union (IoU) measure between predicted tracks and the actual detections, along with the deep feature similarities. The Hungarian algorithm is used for the best assignment of detections and the tracks for accurate correspondences. Finally, the created tracks are continuously updated based on their states, with new states being created when necessary and old ones being deleted to maintain efficiency. The algorithm adapts to changing scenarios while maintaining tracking consistency [46].

### 3.3 Meter per pixel calculation

Controlling the UAV based on the analysis of the input image frame in terms of pixels values could be highly inaccurate. For more precise control of the autonomous UAVs, it is necessary that the distances are in meters. In this section the formula to convert the horizontal and vertical meters per pixel is discussed. The horizontal meters per pixel value can be obtained from Equation 1.

$$\text{Horizontal Meters per pixel}=\frac{2D\tan\left(\frac{H_{\mathrm{FOV}}}{2}\right)}{H_{\mathrm{RES}}}$$ (1)

Similarly, the vertical meters per pixel value can be obtained from Equation 2.

$$\text{Vertical Meters per pixel}=\frac{2D\tan\left(\frac{V_{\mathrm{FOV}}}{2}\right)}{V_{\mathrm{RES}}}$$ (2)

Where, D is the distance between the camera and the object in frame, which we assume to be equal to the altitude measurement obtained from the lidar, HFOV and VFOV are the horizontal and the vertical field of view of the camera respectively, HRES and VRES are the vertical and horizontal resolution of the camera respectively.

### 3.4 Proportional-integral-derivative (PID) controller for precise movement control

**FIGURE 2:**
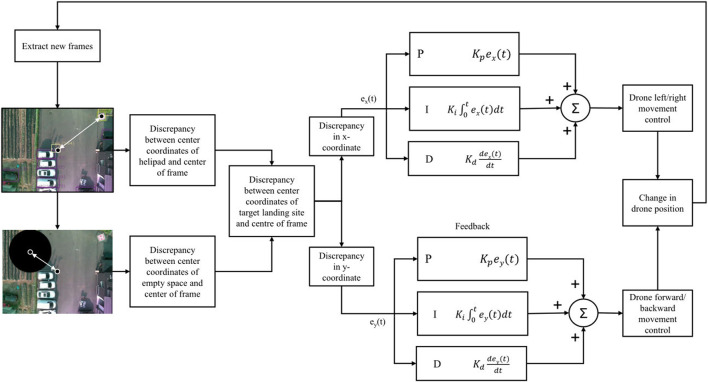
Visualization of the PID controllers in context of the suggested algorithm.

The precise movement of the quadrotor UAV is controlled by two PID controllers. The structure of the two controllers can be visualized in Figure 2. The first controller generates the control values to control the ‘Left/right movement’ using the discrepancy between the x-coordinate of the center of the obtained image frame and the x-coordinate of the center of the landing target. In Figure 2, this controller is placed at the top. The second controller, which can be seen at the bottom of Figure 2, takes in the difference between the y-coordinate of the center of the obtained image frame and the y-coordinate of the center of the landing target to output the control values to control the “forward/backward movement” of the UAV. Throughout the landing, the “yaw” of the drone is constant, and the altitude is slowly decreased by 0.5 m only if the error between the center of the image frame and the landing target is negligible and if there are no obstacles nearby the landing pad.

The overall structure of the two controllers is that of a basic PID controller. The controllers adjust the system output based on the error between the desired setpoint and the actual value of the process variables (Bennett, 2001). For this system, the outputs are the ‘left/right’ and the ‘forward/backward’ movement of the UAV. Furthermore, the desired set points are the center coordinates of the image frames, and the process variables are the center coordinates of the landing target. The error in each step is the difference between the center coordinates of the image frame and the center coordinates of the landing target. The two controllers adjust the “left/right” and “forward/backward” movement utilizing the summation of proportional $\left(K_{p}\right)$ , integral $\left(K_{i}\right)$ and derivate $\left(K_{d}\right)$ components of the error.

The equation of the PID controller for the ‘left/right movement control’ is given by Equation 3.

$$Output_{LR}=K_{P}*e_{X}\left(t\right)+K_{i}*\int_{0}^{t}e_{X}\left(t\right)dt+K_{d}*\frac{de_{x}\left(t\right)}{dt}$$ (3)

Similarly, the equation of the PID controller for the ‘forward/backward movement control’ is given by Equation 4.

$$Output_{FB}=K_{P}*e_{Y}\left(t\right)+K_{i}*\int_{0}^{t}e_{Y}\left(t\right)dt+K_{d}*\frac{de_{Y}\left(t\right)}{dt}$$ (4)

In Equations 1, 2, the $Output_{LR}$ and $Output_{FB}$ are the control values generated by the two controllers for controlling the left/right and the forward/backward movement of the UAV and $e_{x}$ and $e_{y}$ are the error in x-coordinate and y-coordinate respectively.

### 3.5 Finding safe alternate landing space

**FIGURE 3:**
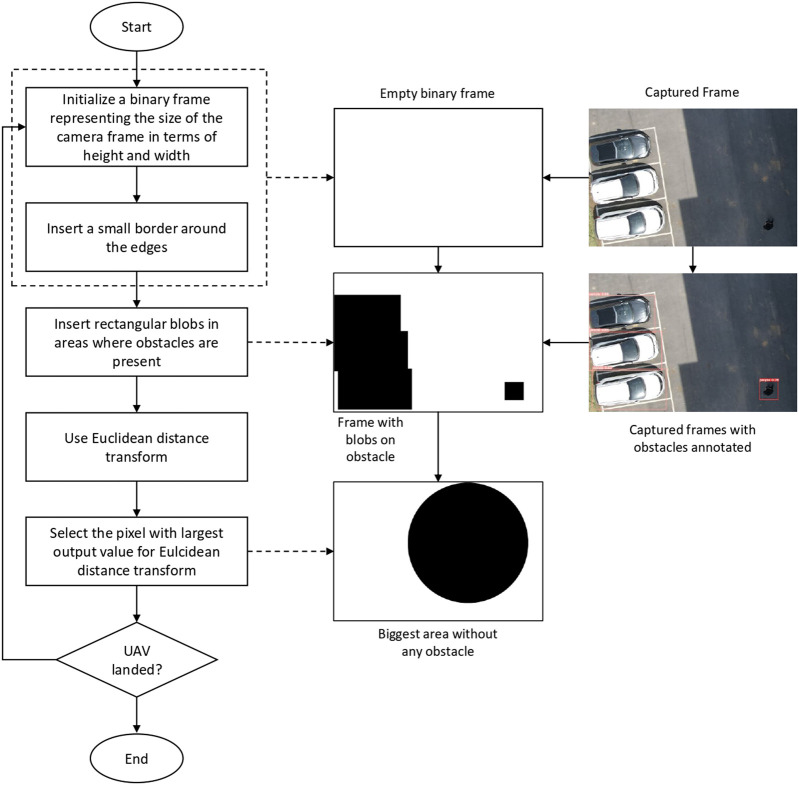
Flow chart for the algorithm to find the safe alternate landing space.

Performing autonomous landing on a landing target using visual cues may not always be a feasible option. The possibility of coming across some undesirable scenarios like the inability to locate the landing target, absence of the visual cues denoting the landing target, and unsafe landing conditions like the presence of other objects near the landing target, makes it necessary for adding a reliable alternate method to perform the landing. We suggest a simple algorithm using input visual cues to find an alternate landing spot without any obstacles near the drone. The overall process is shown in Figure 3.

To find a safe alternate landing space, first an empty image frame with all white pixels and a small border of black pixels is initialized. The size of the initialized image frame is the same as that of input camera frames. Based on the position of the obstacles identified by the YOLOv5 on each input frame, the algorithm draws rectangular black blobs on the initialized image frame where the obstacles are present. Euclidean distance transform (Rosenfeld and Pfaltz, 1966) is applied to the obtained image. The distance transform gives individual values to each pixel of the input frame, which represents the distance from that pixel to the closest black pixel. The biggest output value of the distance transform represents the largest open space in the image frame without any obstacles. The corresponding position of that value represents its center in terms of pixels.

## 4 Setups and experiments

The implementation of the overall method was done in various steps. Initially, a test was performed to check whether the distance in meters calculated by the system is accurate. To compare the performance some existing object detection models, these models were intially trained on a publicly available dataset. Lateron based on this, YOLOv5s model was selected and trained on a self-collected dataset. A simulation of the landing conditions was performed in software before testing the scenarios in real life. This section will provide the details regarding the test to verify the accuracy of the distance calculation, selection of the object detection model, training of YOLOv5s, simulation setup, and the hardware setup.

### 4.1 Test for accuracy of pixels to meter conversion

One of the crucial steps to ensure the autonomous landing is to verify the accuracy of conversions while the algorithm is running. We performed a test to check whether the conversion of distance from pixels to meters is accurate. For this, we took distance measurements between various marked points using a measuring tape. The pictures of these points are taken from the camera in the UAV during a flight, and the altitude of the UAV is continuously recorded for each corresponding frame. Using the images and the corresponding altitude, the distance between the marked points is calculated using the formulae in Section 3.4. The difference between the actual measurements and the calculations is then checked. Table 1 presents the results of this test. The table shows the images captured from the drone, the altitude from which the image was taken, the picture instance of the measurement being taken for the corresponding points, the calculated distance, the measured distance, and the error. It can be verified from Table 1 that the distance calculated in terms of meters based on the images captured from the UAV is accurate enough for the same method to be used in the autonomous landing algorithm.

**TABLE 1:** Results from the test for verifying the accuracy of pixels to meter conversion.

S.N.	Image from UAV	Measurement taken	Altitude	Measured Distance	Calculated Distance	Error
1	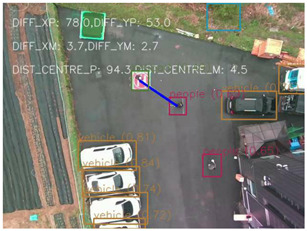	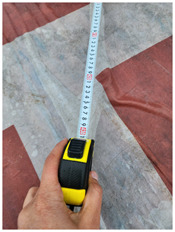	20.0	4.3	4.5	0.2
2	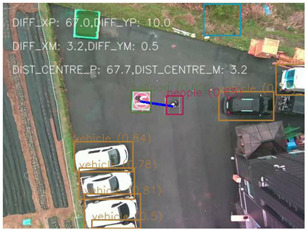	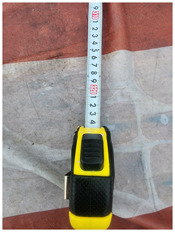	20.0	3.2	3.2	0.0
3	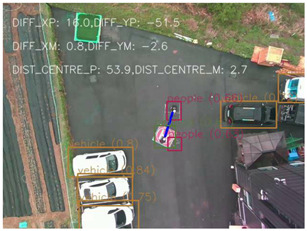	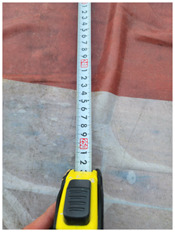	20.1	2.5	2.7	0.2
4	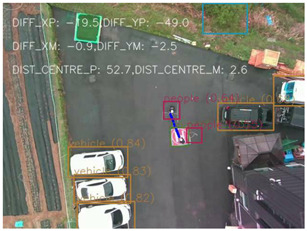	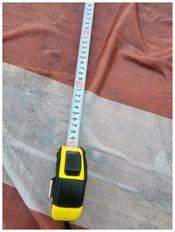	20.0	2.6	2.6	0.0
5	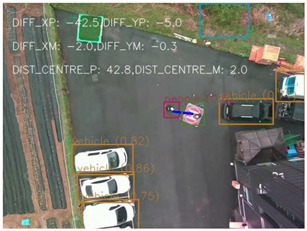	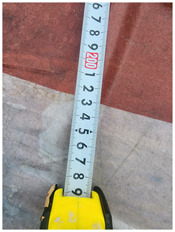	20.0	2.0	2.0	0.0
6	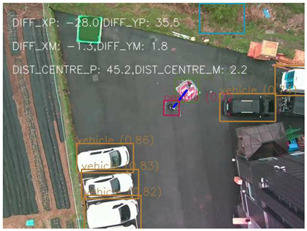	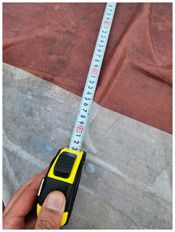	20.0	2.3	2.2	0.1
7	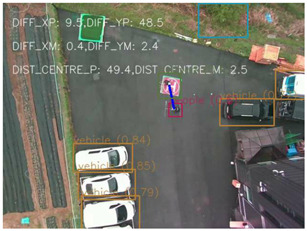	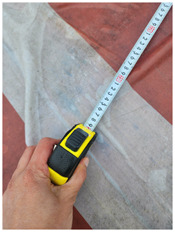	20.1	2.4	2.5	0.1
8	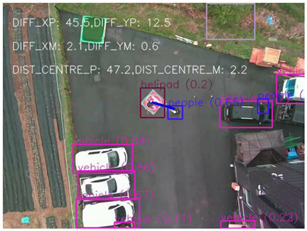	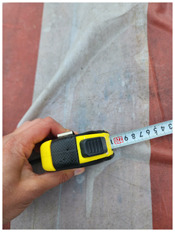	20.1	2.2	2.2	0.0
9	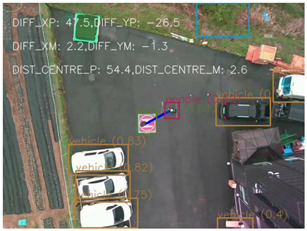	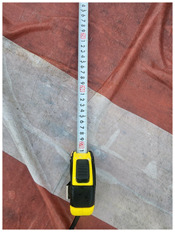	20.0	2.7	2.6	0.1
10	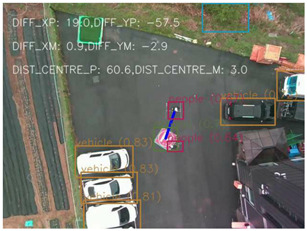	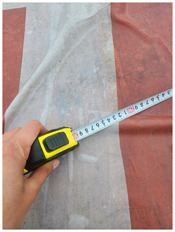	20.0	3.0	3.0	0.0

### 4.2 Selection of object detection model

For this we considered two object detection architectures designed specifically for use in UAV collected images and also the YOLOv5 (Jocher et al., 2020) family. These models were trained on the VisDrone dataset and there performance are compared here. Each of these model were trained using Adam optimizer on a Nvidia RTX 3090 GPU with a batch size of 8 and for 300 epochs. Additionally, these models were run on a Jetson Xavier NX board to compare the number of frames per second (FPS) that can be processed by the device for each of the models. These results have been presented on Table 2. While TPH-YOLOv5 (Zhu et al., 2021) and CMPH-YOLOv5 (Baidya and Jeong, 2022) tend to perform better than rest in terms of precision, recall and mAP, the number of FPS possible to be processed is ideal in YOLOv5s. So we proceed with YOLOv5s model.

**TABLE 2:** Comparison results between different architectures.

Method	P (%)	R (%)	$mAP_{0.5}$ (%)	$mAP_{0.5:0.95}$ (%)	FPS
YOLOv5n	0.36189	0.28197	0.26161	0.13335	**12**
YOLOv5s	0.45251	0.3377	0.33179	0.18063	**12**
YOLOv5m	0.50072	0.37865	0.37837	0.21837	10
YOLOv5l	0.51648	0.39725	0.40004	0.23653	8
YOLOv5x	0.57061	0.39677	0.41358	0.24901	6
TPH-YOLOv5	0.66588	0.54958	0.59177	0.38152	5
CPH-YOLOv5	**0.67406**	**0.55746**	**0.60015**	**0.38612**	5

The bold values represent the best result obtained for that particular metric.

### 4.3 Details of YOLOv5 training

Here we will discuss the details of the training process of the customized YOLOv5 model. This will include the data set used, the evaluation metrics and the results of training.

#### 4.3.1 Dataset

The dataset used in this study was self-collected through various means. A total of 683 images were collected, with most of the pictures taken by the researchers themselves, while some were generated using a generative AI model DALL-E 2 (Ramesh et al., 2022), and the rest are from the web. The dataset is composed of 4 classes: “Helipad,” “People,” “Vehicle,” and “Tree.” The “Helipad” class represents the landing pad and the rest are the obstacles.

#### 4.3.2 Evaluation metrics

The study primarily emphasizes two key performance metrics for evaluating the effectiveness of the proposed model, namely, the Mean Average Precision at intersection over union (IoU) threshold of 0.5 (mAP0.5) and the overall mean Average Precision (mAP). The Mean Average Precision at intersection over union (IoU) threshold of 0.5 (mAP0.5) and IoU threshold ranging from 0.5 to 0.95 (mAP0.5:0.95) are commonly used to evaluate object detection tasks. The mAP measures the average precision of the model across multiple IoU thresholds, which determines the level of overlap required between the predicted and ground-truth bounding boxes to consider them as true positives. The mAP calculates the area under the precision-recall curve for different IoU thresholds, where the precision is the ratio of correctly predicted objects to the total number of predicted object, and the recall is the ratio of correctly predicted object to the total number in ground-truth.

The *mAP* is given by the Equation 5.

$$mAP=\frac{1}{N_{\mathrm{class}}}\overset{N_{class}}{\underset{i=1}{\sum }}AP_{i}$$ (5)

Where $N_{\mathrm{class}}$ is the total number of classes.

Furthermore, $mAP_{0.5}$ only considers the IoU threshold of 0.5, which is a widely used threshold in object detection benchmarks. This metric measures the model’s ability to correctly predict bounding boxes that have an IoU overlap of at least 50% with the ground-truth bounding boxes. The *mAP*_0.5_ is given by the Equation 6.

$$mAP_{0.5}=\frac{1}{N_{\mathrm{class}}}\int_{0}^{1}P\left(R\right)dR$$ (6)

Where $N_{\mathrm{class}}$ is also the number of classes and P and R represent precision and recall respectively at IoU threshold 0.5. Precision and recall are given by Equations 7, 8 respectively.

$$P=\frac{TP}{TP+FP}$$ (7)

$$R=\frac{TP}{TP+FN}$$ (8)

Where TP means true positives, FP means false positives, and FN means false negatives.

Likewise, $mAP_{0.5:0.95}$ considers the IoU threshold in the range of 0.5:0.95. It can be computed by taking the mean of the AP value across all classes and all IoU thresholds between 0.5 and 0.95 with a step of 0.05 (0.5, 0.55, 0.6, 0.7, 0.75, 0.8, 0.85, 0.9, and 0.95).

#### 4.3.3 Results of training

**FIGURE 4:**
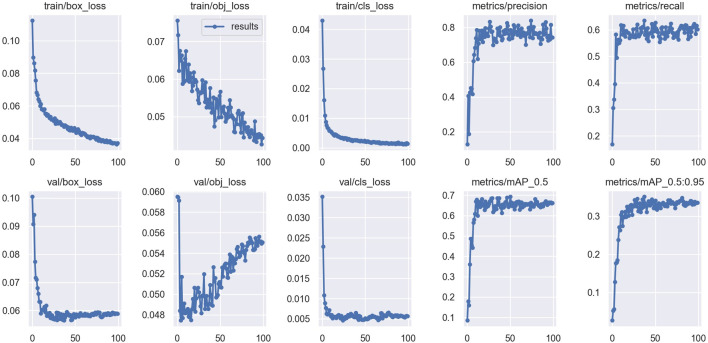
Losses and the metrics of the trained YOLOv5 model. The figure shows the box loss, objectness loss and class loss during training and validation, precision, recall, $mAP_{0.5}$ and $mAP_{0.5:0.95}$ .

The results obtained after training the YOLOv5 model on our dataset have been presented in Table 1. The trained YOLOv5 model obtained a precision value of 0.7498, which means that out of all the objects detected by the model, 74.298% of detection was correct. Furthermore, a recall of 0.60224 was obtained which means that the model was able to detect 60.224% of all the objects present in the test images. Also, the mAP0.5 value of 66.158% and $mAP_{0.5:0.95}$ was obtained. The plots for losses and the metrics of the trained YOLOv5 model is shown in Figure 4.

### 4.4 Simulation setup

**FIGURE 5:**
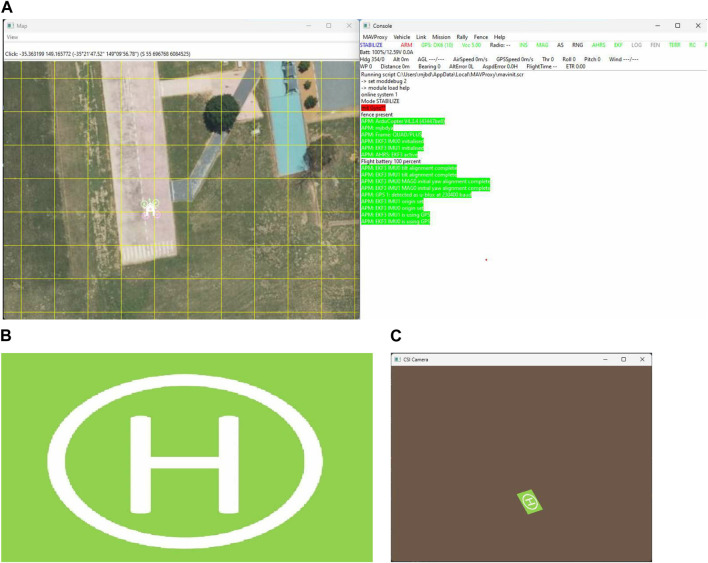
Snippets of different parts of the simulation software: **(A)** Snippet of Ardupilot SITL, **(B)** Input image of the landing pad, **(C)** Simulation of the camera image frame based on the position and orientation of the SITL UAV and the input landing pad image.

The landing simulation was performed using a Python script that converts real-world 3D coordinates to 2D image coordinates and generates a simulated camera frame based on the GPS and UAV orientation information of the UAV simulated on the Ardupilot Software in The Loop (SITL) software. First, an instance of Ardupilot SITL needs to be launched using which the Python script connects to the simulated UAV and continuously receives information like latitude, longitude, altitude, pitch, roll, and yaw of the simulated UAV. An image of the landing area can be passed to the script, and the image can be changed based on the desired scenario to be tested. The script simulates image frames as if they were received from the camera attached to the bottom of the UAV. The position of the landing pad is fixed to a particular location so that it would be visible in the image frames if it was in the field of view of the simulated camera. Figure 5 shows the snippets of the different parts of the simulation performed. In Figure 5, (a) is the snippet of the Ardupilot SITL software, (b) is the input helipad image, and (c) is the simulated output of the camera image frame which is based on the location and orientation of the UAV simulated in SITL software, and the input image of the landing pad. First, both the 3D real-world coordinates of the landing pad are projected onto the 2D image plane of a hypothetical camera attached to the bottom of the UAV and pointed towards the ground. The projected 2D coordinates are matched to the image coordinates, and the frames are rendered by applying perspective transformations. The script continuously receives the UAV position and orientation information and generates the camera frames till the UAV landing is complete.

The written script then controls the position of the simulated UAV based on the simulated frames of the attached camera and the algorithm described in Section 3.5. Additionally, different landing scenarios can be simulated based on the input image of the landing pad. For example, an empty frame without any visual marker denoting a landing pad can be input to simulate the situation where there is no landing pad visible. Similarly, an input image with an object nearby can be input to the software to simulate the scenario of the presence of an unwanted object near the landing pad. In all these conditions, a simulation of the autonomous landing can be performed using Ardupilot SITL and the written Python script. The Pseudo code for this is given by 3.

Algorithm 3 Algorithm for simulating the camera frames based on SITL information.

 **Input:**location of SITL UAV in terms of metres from a reference point **‘vehicle_location’**; attitude of SITL UAV **‘vehicle_attitude’**;landing pad image **‘target’**
 
vehicle_location←location of SITL UAV

 
vehicle_attitude←attitude of SITL UAV

 
target_location←location of landing pad

 **while** the program is running **do**
  
vehicle_location←updated location of SITL UAV

  **vehicle_location_p** ← **vehicle_location** converted in terms of pixels
  
corners← coordinates of corners oftarget

  
new_corners←corners translated in terms of vehicle location and attitude
  
new_corners←new_corners translated in terms of camera frames
  
perspective_transform← matrix for translation of corners to new_corners
  warp perspective of **target** based on **perspective_transform**
  display warped **target**

### 4.5 Hardware setup

**FIGURE 6:**
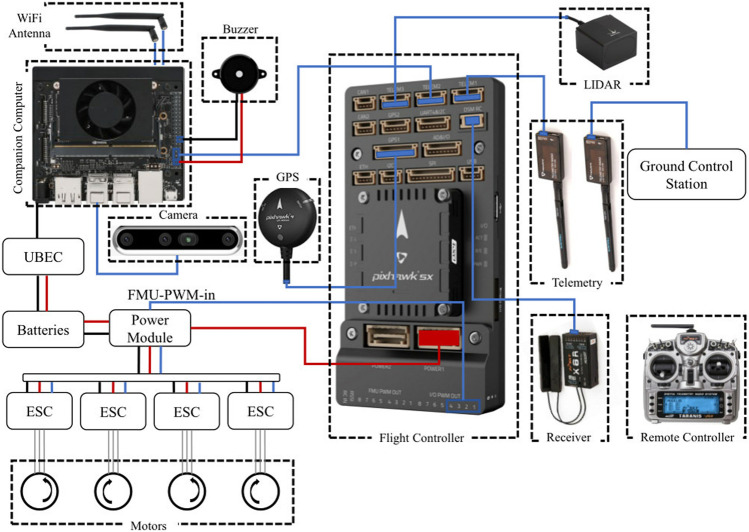
Schematic diagram of the hardware setup.

Figure 6 shows the schematic diagram of the UAV hardware setup. The parts used in the hardware setup are as follows:

• Frame: Quadcopter

• Flight Controller: Pixhawk 5 ×

• GPS: Pixhawk4 GPS module

• Telemetry radio: HoIybro 433 MHz 100 mW

• Propellers: 22-inch, pitch: 11

• Motors: MN605-S kv170

• Controller: Taranis x9d

• FRSky x8r

**FIGURE 7:**
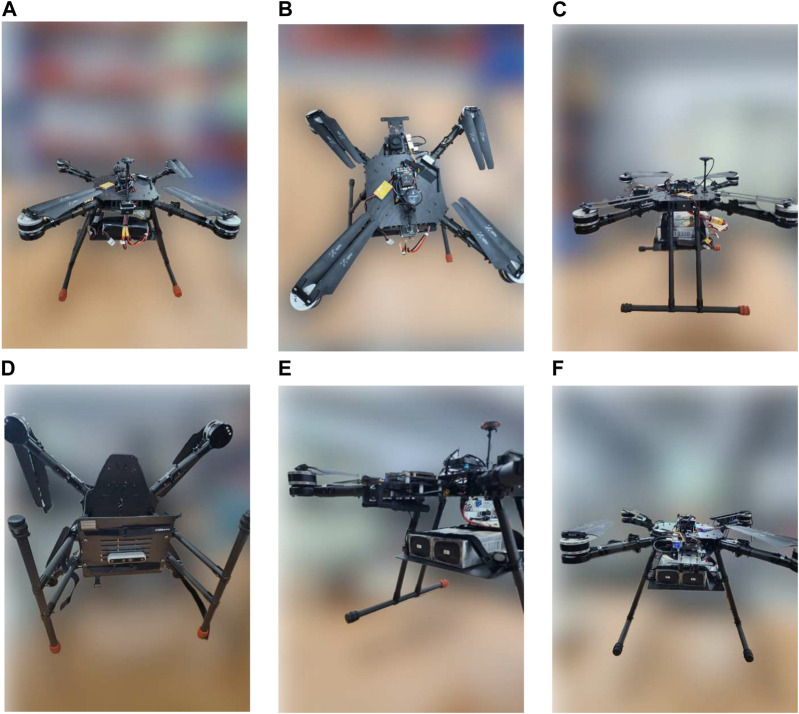
Fully assembled UAV from different fields of view: **(A)** Front view, **(B)** Top view, **(C)** Side view, **(D)** Bottom view, **(E)** Tilted view, **(F)** Back view.

The companion computer, Jetson Xavier NX is where all the computation occurs. The companion computer is loaded with a script to connect to the flight controller Pixhawk 5x, extract images from the Intel Realsense 455D camera, perform the required processing, and then send the control commands to the flight controller. The script loaded on the companion computer also has the YOLOv5 algorithm loaded on it with the trained weights for the recognition of the landing pad and other objects to perform autonomous landing. For getting the accurate altitude of the UAV, a Benewake TF03 lidar is also connected to the flight controller. Human interference may also be necessary during emergencies, so a remote controller is connected to the flight controller using an FRSky x8r receiver. The status of the UAV may be monitored using a ground control station with software like Mission Planner. The UAV after complete assembly can be seen in Figure 7.

## 5 Results of real-life implementation of autonomous landing in different scenarios

**FIGURE 8:**
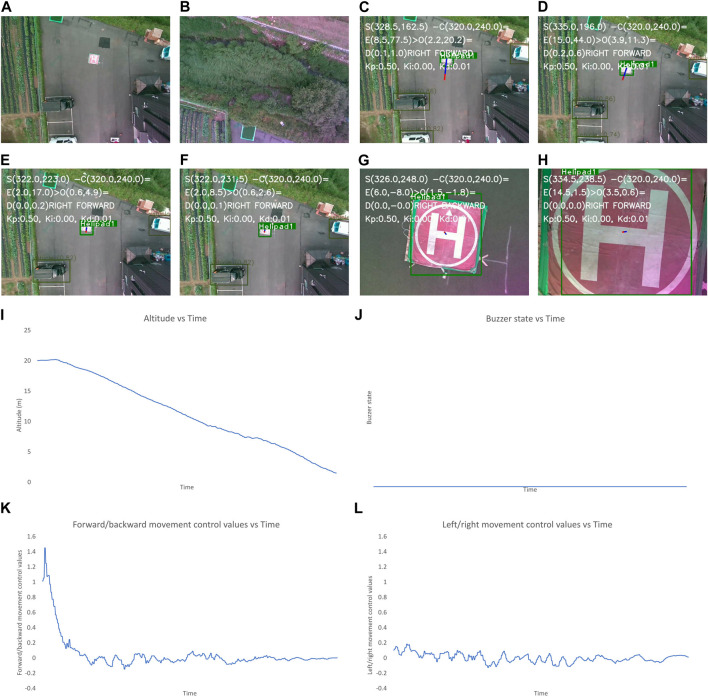
Output for the optimum landing scenario. The images show the **(A–L)** image frames captured by the camera during different states of flights, graphs for the: **(I)** altitude, **(J)** buzzer state, and the control values of: **(K)**, left/right and **(L)** forward/backward movement of the UAV with time throughout landing process. The different state of flight in **(A–H)** are: **(A)** after take-off is completed, **(B)** after first way-point is reached, **(C)** after final way-point is reached and autonomous landing is started, **(E)** while the UAV is moving to make landing pad appear at center, **(F, G)** after the center of frame and position of landing pad have descrepancy of less than 2 m, **(H)** when the UAV altitude is less than 1 m and “LAND” command is sent to flight controller.

**FIGURE 9:**
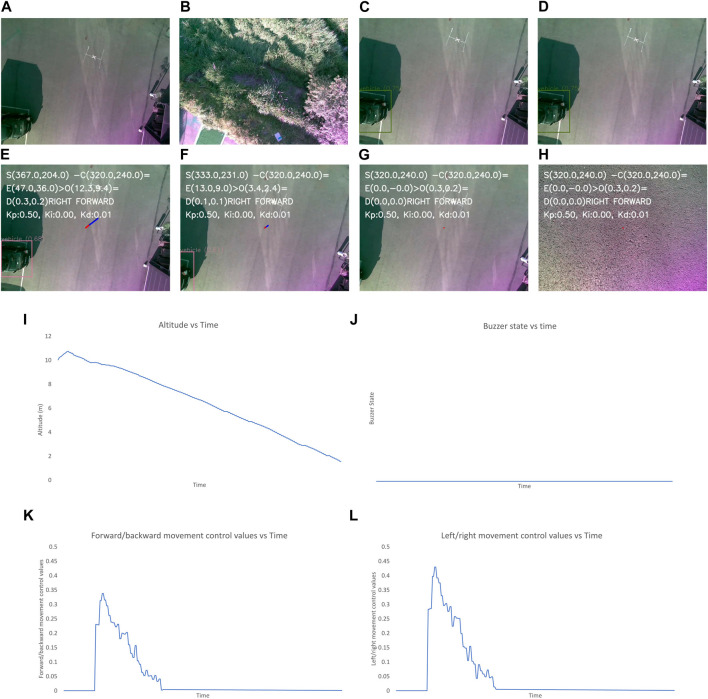
Output when the visual marker for landing pad is not placed. The images show the **(A–L)** image frames captured by the camera during different states of flights, graphs for the: **(I)** altitude, **(J)** buzzer state, and the control values of: **(K)**, left/right and **(L)** forward/backward movement of the UAV with time throughout landing process. The different state of flight in **(A–H)** are: **(A)** after take-off is completed, **(B)** after first way-point is reached, **(C)** after final way-point is reached and autonomous landing is started, **(D)** when landing pad is not detected, **(E, F)** while the UAV is moving to make the empty spot appear at the center, **(G)** after the center of frame and position of empty spot have discrepancy of less than 2 m, **(H)** when the UAV altitude is less than 1 m and “LAND” command is sent to flight controller.

**FIGURE 10:**
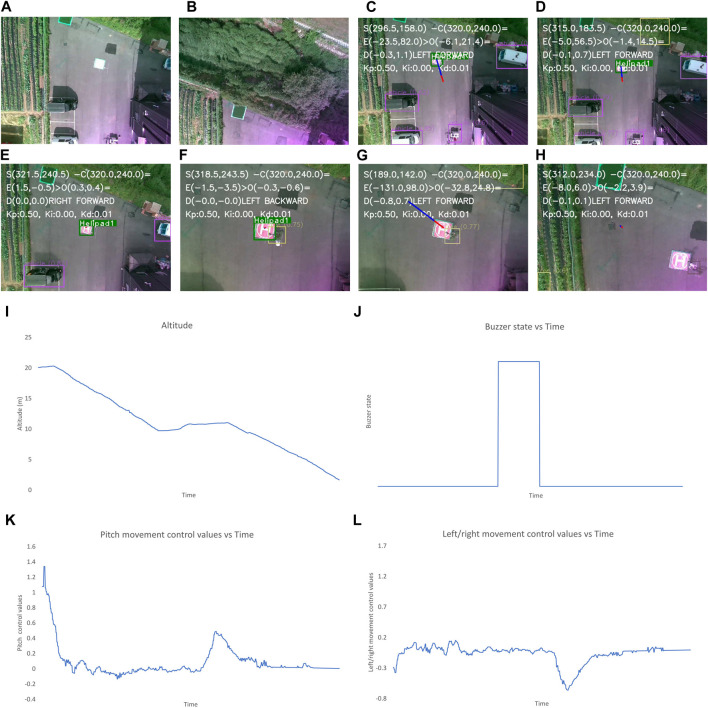
Output when obstacles appear mid-way during the autonomous landing and remain for more than 10 s. The images show the **(A–N)** image frames during different states of flights, graphs for the: **(K)** altitude, **(L)** buzzer state, and the control values of **(M)** left/right and **(N)** forward/backward movement of the UAV throughout the landing process. The different states of flight in **(A–J)** are: **(A)** after take-off, **(B)** after first way-point is reached, **(D)** after final way-point is reached and autonomous landing is started, **(D)** while the UAV is moving to make landing pad appear at center of frame, **(E)** when the center of frame and position of landing pad have discrepancy of less than 2 m, **(F)** when obstacle is detected nearby the landing pad, **(G)** after switching to alternate spot with largest empty space on frame as the target landing spot, **(H)** while the UAV is moving to make the empty spot appear at the center, **(I)** when the center of frame and position of empty spot have discrepancy of less than 2 m, **(J)** after the altitude is less than 1 m and “LAND” command is sent.

**FIGURE 11:**
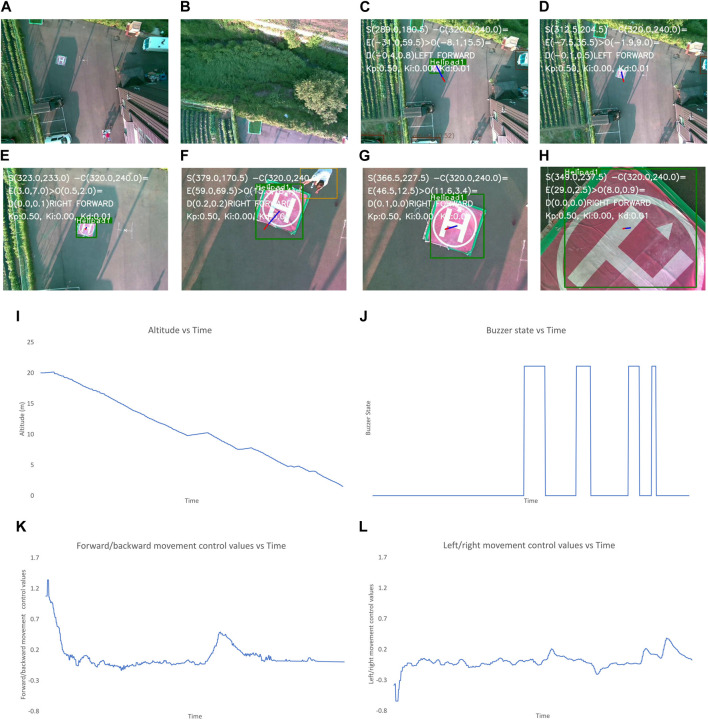
Output when position of landing pad is changed multiple times during landing. The images show **(A–N)** frames captured during different states of flights and graphs for: **(K)** altitude, **(L)** buzzer state, and control values of **(M)** left/right and **(N)** forward/backward movement of the UAV. The different states in **(A–J)** are: **(A)** after take-off, **(B)** after first way-point is reached, **(C)** after final way-point is reached and autonomous landing is started, **(D)** when the UAV is moving to make landing pad appear at center of frame, **(E, F)** after the center of frame and position of landing pad have discrepancy of less than 2 m, **(G)** when the location of landing pad is changed and the UAV should re-positioned, **(H)** when the UAV is moving to make landing pad appear at center of frame, **(I)** after the center of frame and position of landing pad have discrepancy of less than 2 m **(J)** after the UAV altitude is less than 1 m and “LAND” command is sent. Here **(G–I)** happen multiple times so the buzzer runs multiple times, the images are present to make it managable.

In this section, we present the results obtained in various scenarios. First this test, two different ways points are inputted into the UAV mission. The final waypoint is set to be near the position of the landing pad. The script running in the companion computer continuously monitors the state of the mission. Once the mission is completed, the script starts the autonomous landing based on the algorithm mentioned in Section 3.5. The real-life tests of the autonomous landing were performed in the following scenarios:

• The first scenario is the most optimum landing condition when the landing pad is clearly visible once the mission is completed and there are no obstacles nearby the landing pad throughout the landing process. The results for this have been shown in Figure 8.

• The second scenario is created by not placing any visual markers that denote the landing target. The results for this have been shown in Figure 9.

• The third scenario is created by placing a plant and a human near the landing pad after it has been a while since the landing has started. The results for this have been shown in Figure 10.

• The final one is created by moving the position of the visual markers denoting the landing pad multiple times during landing procedure. The results for this have been shown in Figure 11.

The results shown in Figures 8–11 include the image frames of key moments captured from the camera, the graphs of altitude, control values sent for the left/right and forward/backward movement of the UAV, and the buzzer state throughout the landing process for the four scenarios.

In Figure 8, the altitude continuously decreases due to optimum conditions and the buzzer state is also constantly low due to the absence of obstacles near the landing target. The control values for the left/right movement seem erratic and change continuously, but these values are small, so the system is still very stable. The graph for the forward/backward movement control values is smooth, indicating smooth control of the UAV forward/backward movement.

In Figure 9, the altitude of the UAV initially seems to be increasing, which indicates that the landing pad is not identified after the completion of the mission. Ten seconds later, the algorithm deviates the UAV to land in an alternate safe spot. Subsequently, the altitude gradually decreases. During the initial phase where the landing spot is not identified, the control values are generated for the left/right and forward/backward movement of the UAV.

In Figure 10, initially, the altitude is gradually decreasing however, after a while a constant altitude is maintained. At this point, obstacles are present near the landing pad. During this period the buzzer state is also high. Even after a certain period, the obstacle is still present, so the UAV is maneuvered toward an alternative landing spot. During this, the control values for the forward/backward and left/right movement initially change drastically since the safe landing spot without obstacles must be farther away from the UAV position because of the present obstacles.

In Figure 11, the altitude is constantly decreasing except for a few periods where the UAV maintains a constant altitude. During this, a human changes the position of the landing pad, and the human appears as an obstacle. The buzzer states are also high during these periods.

## 6 Discussion and conclusion

In recent years, the usage of autonomous UAVs has seen a significant rise in various applications, such as aerial photography, surveying, and monitoring. One of the critical aspects of autonomous UAVs is their ability to perform autonomous landings even in adverse conditions of the real world. In this paper, we present a system for vision-based autonomous landing system with robust capabilities to perform autonomous landing in real-world undesirable scenarios like the inability to detect the designated visual marker or the absence of the marker altogether, the presence of multiple such markers, and the presence of obstacle nearby the marker. The proposed landing system encompasses multiple key algorithms that are integrated to collectively strengthen the system’s performance. The integration of version 5 of You Only Look Once (YOLOv5), DeepSORT, Euclidean distance transform, and a Proportional-Integral-Derivative (PID) controller forms the foundation of this robust autonomous landing solution. YOLOv5 is employed to address the task of identifying both the designated landing area’s visual marker and potential obstacles such as pedestrians, vehicles, and trees. The DeepSORT algorithm plays a vital role in tracking identified objects. The utilization of Euclidean distance transform in conjunction with object detection provides the system with the ability to discern open spaces devoid of obstacles within the designated landing area. The PID controllers form the control strategy of the system, generating precise movement commands for the UAV based on the visual cues of the target landing area and the detected obstacles. This controller ensures smooth and controlled maneuvers, enhancing the accuracy of the landing procedure. To establish the efficacy and safety of the proposed system, a comprehensive approach to testing and validation is adopted. Initial tests are conducted to evaluate the system’s functionality, followed by a software simulation to further analyze its performance in a controlled environment. This stepwise validation strategy mitigates potential risks and allows for refining the system before real-world tests. Subsequently, a hardware system is developed, incorporating the autonomous landing system, and rigorously tested in real-life scenarios. The hardware testing validates the feasibility of implementing the proposed solution in practical applications and offers insights into its performance under dynamic conditions. In conclusion, this paper presents a comprehensive and innovative autonomous landing system tailored for quadrotor autonomous UAVs. The integration of YOLOv5, DeepSORT, Euclidean distance transform, and a PID controller forms a synergistic approach to address the challenge of precise landings in varying conditions, including the presence of obstacles and the absence of visual markers. The system’s effectiveness is established through a rigorous testing process, which encompasses initial functional tests, software simulations, and real-life hardware testing. The outcomes of these tests demonstrate the system’s ability to successfully navigate complex landing scenarios, confirming its robustness and reliability. The presented autonomous landing system holds significant potential for enhancing the autonomy and adaptability of UAVs in critical missions, including search and rescue, surveillance, and package delivery. As UAV applications continue to expand, the advancement of such autonomous landing solutions becomes pivotal for ensuring safe and efficient autonomous operations.

## Data Availability

The raw data supporting the conclusions of this article will be made available by the authors, without undue reservation.
